# Case of Fracture of the Scull

**Published:** 1790

**Authors:** George Wilkinson

**Affiliations:** Surgeon at Sunderland, Member of the Royal College of Surgeons, and honorary Member of the Chirurgo-physical Society of Edinburgh


IX. *Cafe of Fracture of the Scull. Communicated in a Letter to Dr.* Simmons, *F. R. S. by Mr.* George Wilkinfon, *Surgeon at Sunderland, Member of the Royal College of Surgeons, and honorary Member of the Chirurgo-phyfical Society of Edinburgh.*

ON Monday, the 19th of Auguft laft, I was fent for to John Goddard, a boy fix years of age, who had been kicked immediately above the orbit of the left eye by the heel of a horfe's fhoe. The wound was tranfverfe, tranſverſe, about half an inch in length, and bled pretty freely; upon introducing my finger into it, I could plainly diſcover that the ſcull was fractured, and much depreſſed.

As the boy was free from any alarming ſymptoms, I contented myſelf at that time with dreſſing the wound with a little dry lint.

The day following I called, and found him in good ſpirits, complaining only of a little ſickneſs, occaſioned by a purging mixture he had taken that morning.

Friday, the 21ſt, in conſultation with Mr. Akenhead, ſurgeon of this town, I proceeded to enlarge the wound. The inciſion was carried upwards, and the ſcalp diſſected backwards towards the temporal muſcle, by which means we diſcovered the fracture with conſiderable depreſſion. The ſize of it correſponded to the heel of the horſe's ſhoe. The trephine being applied ſo as to include a ſmall portion of the fracture, ſeveral pieces of bone were extracted, and a diſcharge given to ſome blood extravaſated on the dura mater; the dura mater itſelf appeared to be quite free from injury. The wound was dreſſed with lint ſpread with cerate, and the flaps of the ſcalp were left looſe upon it.

 Three

Three days after this he was dreſſed, and a larger piece of bone that had been greatly depreſſed (and which could not well be diſcovered at the firſt dreſſing on account of the extravaſated blood) was extracted.

Finding the whole of the depreſſed bones extracted, and the dura mater free from injury, I now brought forward the flap ſo as nearly to cover the parts from which it was ſeparated. The edges of the wound were dreſſed with narrow pieces of lint ſpread with cerate, and the parts brought as nearly as poſſible in contact with each other, and retained by narrow ſlips of adheſive plaſter of a ſufficient length. Theſe dreſſings were retained by a double-headed roller applied with gentle preſſure.

In the tract or courſe of the inciſions the edges of the wound digeſted kindly, and a ſmall quantity of matter could now and then be preſſed out from under the ſcalp. The diſcharge continued every day to become leſs and leſs, and in the courſe of a month the whole was (excepting the vacuities of the fracture not being quite filled up) perfectly healed.

I am aware that union by the firſt intention in caſes of fractured ſculls, or where the trephine is obliged to be applied, cannot, in general practice, practice, always prove succesful; yet in such favourable situations as that of my patient very little doubt need be entertained of its answering the desired purpose. Much, however, will depend upon the judgment of the surgeon, as well as the nature and situation of the injury received.

To point out the advantages of this mode of treatment, as recommended by Mr. Mynors in his late publication on this subject, would be altogether superfluous, as every person ever so little conversant with accidents of this sort well knows that depriving the cranium of its covering is often the cause of a long and tedious cure, which very rarely takes place independent of exfoliation.

These and such like considerations, as well as my wish of making this mode of treatment more generally known, have induced me to offer it, Sir, to you for insertion in your useful publication.

*Sunderland,*
October 31, 1790.

X. *Some*

